# Outcomes of Intramuscular Gluteal Augmentation With Implants Using Tumescent Local Anesthesia

**DOI:** 10.1007/s00266-023-03342-x

**Published:** 2023-04-17

**Authors:** Emilio Trignano, Matilde Tettamanzi, Corrado Liperi, Edoardo Beatrici, Pietro Luciano Serra, Claudia Trignano, Corrado Rubino

**Affiliations:** 1 Department of Surgical, Microsurgical and Medical Sciences, Plastic Surgery Unit, University of Sassari, Sassari, Italy; 2 Intensive Care Unit, Emergency Department, AOU Sassari, Sassari, Italy; 3 Department of Urology, Humanitas Research Hospital – IRCCS, Milan, Italy; 4 Department of Biomedical Sciences, University of Sassari, Sassari, Italy

## Abstract

**Background:**

Tumescent local anesthesia (TLA) describes the practice of injecting a very dilute solution of local anesthetic combined with epinephrine and sodium bicarbonate into the tissue until it becomes firm and tense to obtain local anesthesia and vasoconstriction. The use of TLA in augmentation intramuscular gluteoplasty has never been described for implants positioning. Advantages of the TLA technique include a reduction in blood loss through epinephrine-induced vasoconstriction and hydrostatic compression from the tumescent effect. We describe TLA technique for primary intramuscular gluteal augmentation, reporting our experience during the last 5 years.

**Methods:**

From 2017 to 2021, 20 patients underwent bilateral primary gluteal augmentation under TLA and conscious sedation. The tumescent solution was prepared with 25 mL of 2% lidocaine, 8 mEq of sodium bicarbonate, and 1 mL of epinephrine (1 mg/1 mL) in 1000 mL of 0.9% saline solution. The solution was infiltrated with a cannula inside the gluteus maximus muscle intra-operatively.

**Results:**

The mean age of the patients was 39, 15 years. The average amount of tumescent solution infiltrated was 240 mL per gluteus. Operating time was 1 h and 40 min, and recovery room time averaged 240 min. Major surgery-related complications were found in 15% of patients (2 hematomas and 1 seroma) and minor complications were described in a total of 8 patients (4 wound dehiscence and 1 dystrophic scar formation). No signs of adrenaline nor lidocaine toxicity were reported and conversion to general anesthesia was never required.

**Conclusions:**

The tumescent local anesthesia technique represents a safe and efficacious technique for performing gluteal augmentation surgery with an intramuscular implant positioning. The advantages of this technique are safety, reasonable pain control during and after surgery and a low incidence of postoperative side effects due to general anesthesia avoidance.

**Level of Evidence IV:**

This journal requires that authors assign a level of evidence to each article. For a full description of these Evidence-Based Medicine ratings, please refer to the Table of Contents or the online Instructions to Authors www.springer.com/00266.

## Introduction

Body contouring has indeed been a part of the feminine beauty standard in many cultures throughout history. The breast and buttocks have often been seen as important areas for shaping and enhancing in order to achieve the desired aesthetic. In recent years, there has been a rise in gluteal augmentation surgery demand. There are a variety of different techniques that can be used to improve the contour of the gluteal area. Some of these techniques involve liposuction, which creates a slimmer, more sculpted appearance. In some cases, fat can be grafted into the gluteus to create a fuller shape. Implant placement have developed in recent years and although gluteal augmentation has a high rate of patient satisfaction, the rate of complications is still high [1]. Implants visibility and palpability has mostly been associated with subcutaneous and subfascial placement, which can provide satisfactory postoperative recovery but at the same time confer a limited soft tissue coverage of the implant [2, 3]. The introduction of submuscular implant placement for buttocks augmentation has been associated with several benefits [4]. Implants are placed under the muscle, they are less visible and can create a more natural-looking appearance. This can also help to reduce the risk of complications such as implant malpositioning, displacement, and extrusion. Undesirable shaping of the implant and the gluteal area, however, is sometimes observed in some groups of patients [5]. Nonetheless, there is a growing trend toward the use of intramuscular implant placement [6, 7]. These studies have suggested that this approach may produce better results compared to other gluteal augmentation techniques such as subcutaneous, subfascial or submuscular implants placement. Moreover, we have found that satisfactory outcome and good results can be achieved in selected patients after intramuscular augmentation combined with autologous fat grafting.

At surgery, the choice of local or general anesthesia depends on many factors and on the preferences of the patient and the surgeon. Tumescent local anesthesia (TLA) consists in the infiltration of large volumes of saline solution with lidocaine and epinephrine in the muscular compartment [8–10]. With this technique, the time of surgery is slightly longer, but intramuscular dissection is facilitated, and bleeding and postoperative pain are reduced. The use of TLA technique was developed in liposuction but is also routinely used for several other surgeries. To date, it had never been described for gluteoplasty.

We propose a surgical technique for placing the implants in an intramuscular position using the TLA technique, which can lower the most common complications, solve technical difficulties, and offer outcomes that are not always achieved through other methods.

## Materials and Methods

This study evaluated patients’ medical records who underwent augmentation gluteoplasty between 2017 and 2021. Every patient’s indication for surgery was an aesthetic correction for gluteal hypoplasia, performed in two accredited outpatient clinics. All procedures were carried out following standard anesthesiology protocols. A board-certified anesthesiologist was part of the surgical team together with a board-certified plastic surgeon, an assistant surgeon, and an operating room nurse.

The patients were fully informed about implant-based gluteal surgery use, indications, and possible complications (i.e., implant dislocation and postoperative bleeding) and consciously consented to the surgery. The preoperative exams included routine blood checks, and cardiac examinations, and patients were selected according to the following inclusion criteria: American Society of Anesthesiologists (ASA) status I or II. Nonetheless, exclusion criteria were ASA status III or more, pregnancy, BMI > 35 or < 18, and local anesthetic allergy.

**Fig. 1:**
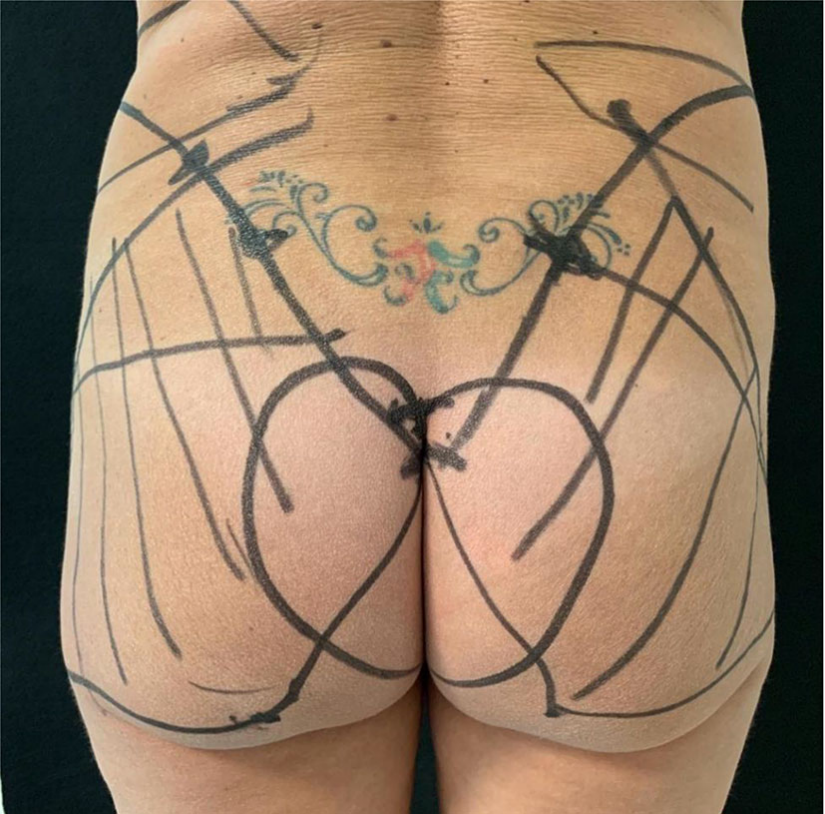
Preoperative markings. The intergluteal fold is marked. Joining the coccyx to the ischial tubercle and continuing up to the femur, we indicate the full size of the gluteus maximus muscle and, therefore the space where the intramuscular gluteus implant will be placed. Of notice is the area of suprafascial detachment on both sides of the intergluteal fold measuring 6 cm on each side with an inverted heart shape

Specific implants for the gluteus were used. We mainly implanted round microtextured highly cohesive prostheses (Polytech, Nagor). The volumes of the implants ranged from 240 to 390 cc. Implant selection must be made before the surgery because no sizer can be used during the procedure. Antiplatelet medications were stopped 5–7 days before surgery or changed to acceptable alternatives. Initially, the patient was marked preoperatively in the upright position, and photographs were taken (Fig. 1). Firstly, we identify the point of the skin incision: the intergluteal fold. The first marking was the upper limit of the incision, which remains in the intergluteal fold and totally disappears when the patient stands. Secondly, the sacrum-iliac joint was marked, and immediately lower and medial, we found the posterior iliac spine. From that point, we proceeded toward the iliac crest, which was in a higher position and indicates the insertion of the gluteus maximus. From the iliac spine, we marked a point at 5 cm going up along the iliac crest. The head of the femur was identified and joined with that point, resulting in a line corresponding to the superior margin of the gluteus maximus. Two other essential marking points were the ischial tubercle and the coccyx. We drew a line joining the coccyx to the ischial tubercle and continuing up to the femur, indicating the full size of the gluteus maximus muscle and, therefore the space where the intramuscular gluteus implant will be placed. It is important to draw an area of suprafascial detachment on both sides of the intergluteal fold because the incision must not be directly made in the gluteus muscle. Still, a first detachment above the gluteus maximus fascia must be performed. This area measured 6 cm on each side of the intergluteal fold and had an inverted heart shape.

**Fig. 2:**
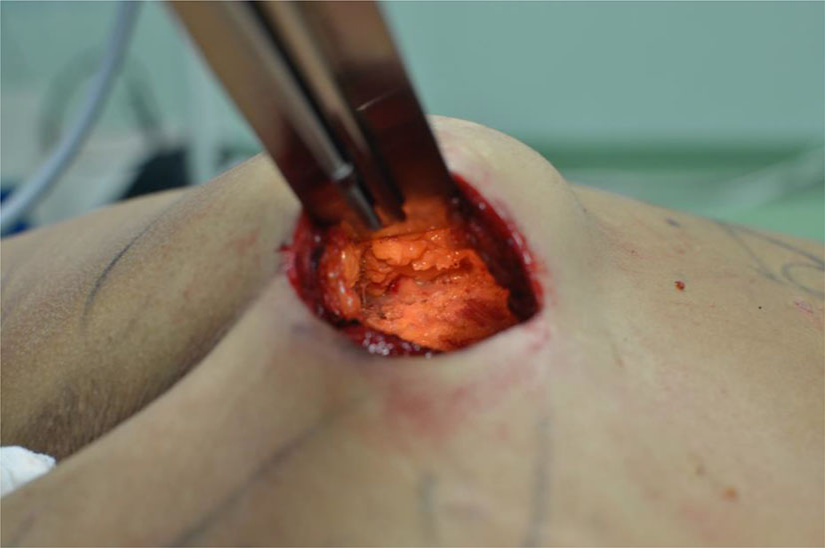
Gluteus maximus and its fascia are exposed

**Fig. 3:**
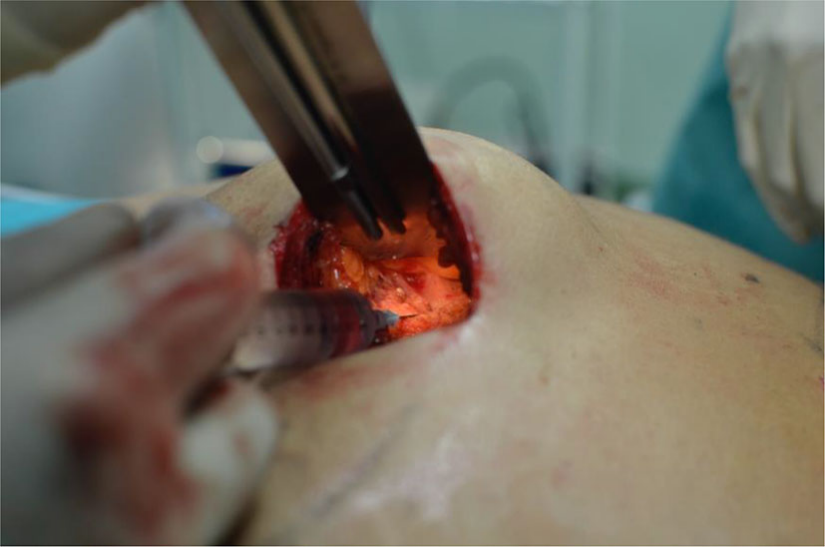
A small lidocaine bolus is performed to incise the muscle for the cannula positioning

**Fig. 4:**
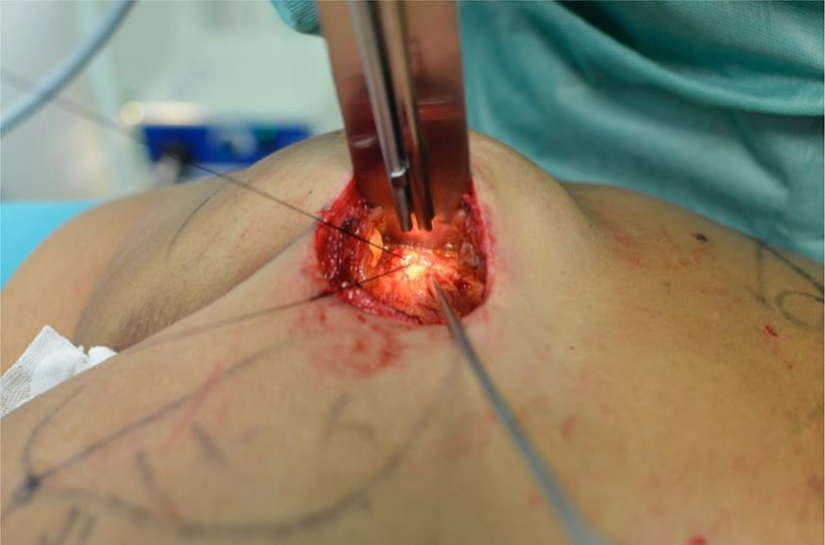
A cannula is inserted within the muscle, connected to a peristaltic infiltration pump, and positioned in this plane for the TLA infusion

**Fig. 5:**
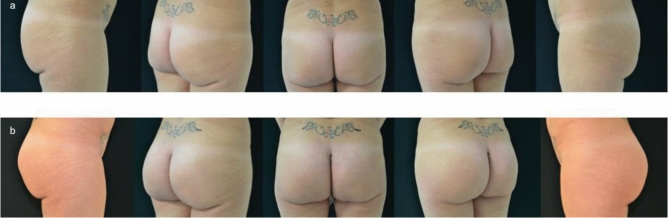
**a** Preoperative view. **b** Postoperative view after 6 months

**Fig. 6:**
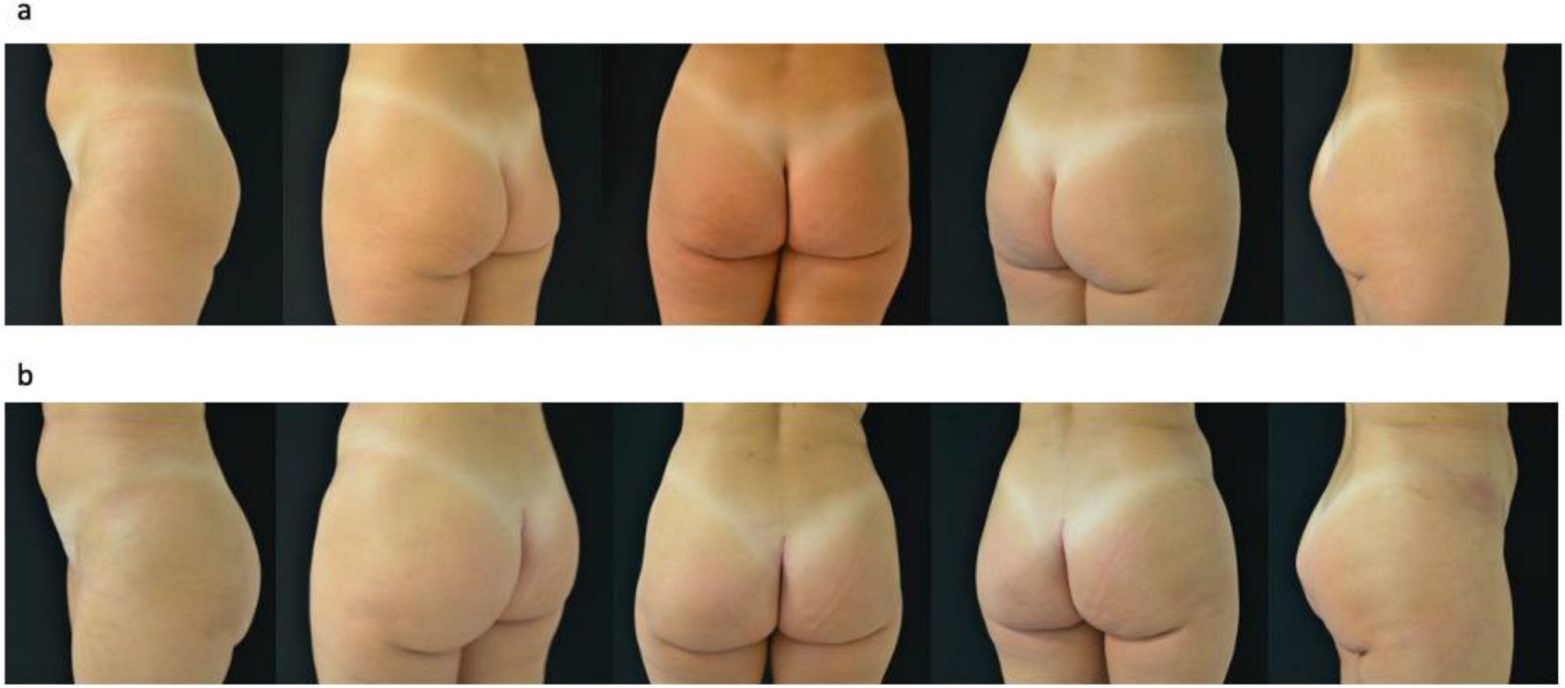
**a** Preoperative view. **b** Postoperative view after 1 year

Before the gluteal augmentation we performed liposuction in the lumbar region bilaterally. The fat graft was harvested from the lumbar region using a 4-mm cannula with beveled 0.5-mm ports connected to the PureGraft© System 150 mL and the suction system LipoSurg©. The autogenous fat grafting was purified and filtered using lactated Ringer solution through the closed system and transferred to 60-mL Luer-Lock syringes for injection [11]. After local anesthetic infiltration, the surgery began with a 1 cm spindle-shaped skin incision in the intragluteal sulcus with reference to the skin markings performed preoperatively. This was followed by de-epithelization of this area which represents the sacro-cutaneous ligament. This structure must be carefully skeletonized without altering or damaging it to allow the reconstruction of the intergluteal fold. The incision was then deepened until the gluteal fascia was reached, leaving it intact. The suprafascial detachment of the inverted heart proceeded up to the external margin using an electrosurgical knife. At this point, the gluteus maximus and its fascia were exposed (Fig. 2). Gluteus anesthesia was performed with a tumescent solution: 25 mL of 2% lidocaine, 8 mEq of sodium bicarbonate, and 1 mL of epinephrine (1 mg/1 mL) in 1000 mL of 0.9% saline solution. Overall, 240 mL were introduced per gluteus. A cannula was inserted within the muscle after a small lidocaine bolus was performed (Fig. 3), connected to a peristaltic infiltration pump, and positioned in this plane (Fig. 4). The tumescent local anesthesia guaranteed complete anesthetization by direct contact. The first incision was made 20 min later to allow epinephrine and lidocaine to have their effect. In patients with low body weight, less tumescent solution was needed to obtain the anesthetic effect and to prevent reaching drug toxicity levels. The fascia was incised in the direction of the muscle fibers, and the incision of the gluteus maximus muscle was performed 3 cm into the muscle, parallel to its fibers. The intramuscular plane was then dissected, and we used the distal phalanx of the fingers as an approximative reference measure. The maximum thickness of the gluteus maximus muscle, between 5 and 7 cm, tends to be standard. During the detachment, it was essential to press down the muscular plane because it tended to heave with the risk of breaking the fibers and the consequence of making the prosthesis too visible after a few months. We proceeded with the detachment up to the limits of the muscle according to the preoperative design. An implant pocket of the size necessary to accommodate the implant was created. Blood vessel coagulation was performed progressively during dissection before insertion of the prostheses to prevent secondary bleeding after clearance of the vasoconstrictive effects of adrenaline. Before implant positioning, sterile drapes and gloves were changed. Surgical drains were not used. After implant positioning, the overlying muscle should completely close and cover the prostheses if correctly positioned. Sequential planes closure was performed using Nylon 2-0 for the muscular pocket. The intergluteal sulcus was reconstructed with a suture linking the two flaps to the underlying ligament, Monocryl 3-0 for the subcutis and Nylon 5-0 for the skin. The wound was covered with a sterile dressing. Gluteoplasty was completed with lipofilling. After 4 h of observation, patients were discharged. After surgery, patients wore an elastic compressive band for 4 weeks. According to allergy status, an oral antibiotic (amoxicillin 875 mg/clavulanic acid 125 mg or ciprofloxacin 500 mg twice a day) was prescribed for 5 days, and postoperative controls were planned after 1 day, 1, 2 weeks, 1–3–6 months, and 1 year (Fig. 5, 6).

## Results

During 5-years, 20 patients underwent bilateral intramuscular gluteal augmentation. Patient ages ranged from 25 to 56 years, with a mean age of 39.15 years. All patients were females. The mean body weight was 58.05 kg, and the mean BMI was 21.9. All surgical procedures were performed using the TLA technique. The average amount of tumescent solution infiltrated was 240 mL (200–280 mL) per gluteus, and we reported no signs of adrenaline or lidocaine toxicity. Moreover, conversion to general anesthesia was never required. A significant increase in the gluteal volume was obtained, maintaining a natural and harmonious aesthetic result. The average surgical time using the TLA technique was 1 h and 40 min. This time included bilateral infiltration, waiting time, and surgical procedure until completion. The waiting time from infiltration to muscle dissection was 20 min. Starting the dissection before 20 min resulted in pain in most patients, whereas waiting longer provided no additional benefits for the patient. All patients reported no pain during skin cutting or gluteal muscle splitting. Implant size ranged from 240 to 390  cc. Among the major postoperative complications, we reported 2 cases of hematomas and 1 seroma (no one requiring reoperation). The minor complication rate was 40% (8/20), represented by 6 cases of wound dehiscence and 2 cases of dystrophic scars. The tension in the intergluteal fold is high and we observed wound dehiscence despite a careful skeletonization of the sacro-cutaneous ligament.

We had no cases of implant dislocation (Table 1). Patients were eventually satisfied with the TLA procedure and did not report any discomfort during the intra-operating infiltration or the complete surgical procedure. Moreover, most patients were satisfied with the esthetic results 1 year after surgery, and satisfaction was evaluated by addressing a survey 3 months after surgery (Table 2). The survey was the same test we used for a previous study conducted in our center for patients who underwent submuscular breast augmentation using tumescent local anesthesia [12]. Patients were asked to rate the pain management and satisfaction of the esthetic result from ‘‘unsatisfactory’’ to ‘‘outstanding”. Patients mainly were highly satisfied. Those who were unsatisfied were the ones who experienced minor complications. All our patients had a follow-up of at least 24 months.

**Table 1:** Complication rate after gluteal augmentation in 20 patients.

Complications	Patients	%
Hematoma	2	10
Seroma	1	5
Implant dislocation	0	0
Wound dehiscence	4	20
Dystrophic Scar	1	5
Need for reintervention	0	0

**Table 2:** Satisfaction survey at 3 months follow-up.

Scale	Patients	%
Outstanding	7	35
Excellent	6	30
Good	4	20
Satisfactory	2	10
Unsatisfactory	1	5

## Discussion

This article presented our experience of 20 consecutive cases of TLA intramuscular gluteal augmentation over 5 years. Our postoperative complication rate was 40% between major and minor complications, but we had no cases of reintervention. Conversion to general anesthesia was never necessary, and no adverse events during TLA were recorded [12]. Before us, none ever described intramuscular gluteal implant in local anesthesia. Nowadays gluteoplasty is still a procedure primarily performed under general anesthesia. Gluteal augmentation has been performed since 1969 as one of the first procedures used to remodel and improve the gluteal region. The original report by Bartels et al. [13] first described the gluteal implantation technique with breast implants, resulting in several major complications such as dislocation, asymmetry and capsular contracture.

Since then, many techniques for augmentation gluteoplasty have been proposed, mainly differing concerning implant location. Following Bartels’ work, Gonzalez-Ulloa [2] described subcutaneous augmentation in 1970, Robles et al. [4] presented a technique involving the insertion of implants in the submuscular space in 1984, De la Pena [3] employed a subfascial approach (1990). Eventually Vergara [6] and Gonzalez [7] described an intramuscular technique.

A study by Aslani and Del Vecchio [14] described in 2019 the use of a water jet technology to obtain hydrodissection of the muscular pocket. They performed two paramedian incisions approximately 1 cm lateral to the coccyx to access the gluteus, incised the gluteal fascia perpendicular to the direction of the gluteus muscle fibers, and dissected through the muscle to a depth of 2–4 cm. Once this thickness was reached, they advanced a high-pressure water jet tumescent infusion cannula parallel to the gluteus maximus muscle fibers in a blunt manner, to establish the proper depth of the intramuscular plane. In a fan-like pattern, the cannula was used to hydrodissect the muscle fibers and to vasoconstrict small intraseptal vessels, setting up ensuing blunt dissection.

Del Vecchio’s technique eventually used progressive instrument dissection to precisely define the correct intramuscular plane of the implant pocket. At the same time, we preferred hydrodissection by TLA infusion, electrocauterization, and used a dissector and a duckbill-type spreader to prepare the pocket. Dissecting too superficially leaves insufficient muscle coverage over the implant; dissecting too deeply leads to potential damage to deep structures. Our technique is safe, and by progressively checking with the distal phalanx during the dissection, assures the creation of a pocket at the ideal muscle depth. Lastly, Del Vecchio performed fat transplantation after pocket dissection and before the final implant placement. We prefer to inject fat after implants positioning, in a way we are perfectly sure where the prostheses are positioned and where fat injection must be safely performed. Moreover, our technique reduces fat trauma from implants positioning. Another interesting article by Del Vecchio [15] evaluates the combination of intramuscular and submuscular implants positioning techniques. They presented a novel dual-plane pocket dissection to overcome the limitations of intramuscular and submuscular techniques and combine the benefits that both have. The dual plane pocket means a submuscular plane in the cranial half of the pocket and switching to an intramuscular plane in the caudal half. In our opinion, this technique is more complicated than intramuscular positioning, and carries high risk of bleeding and sciatic nerve dissection. For this reason, we still prefer our technique for its easiness in performing gluteus maximus coverage of the implant and safeness. Petit and Badiali [16] presented a series of 100 cases of submuscular biconvex buttock implants. They consider this technique safe and reliable, carrying the benefit of perfectly covering, protecting, and hiding the implant, making it almost impalpable and invisible. We know from the literature that sometimes submuscular positioning of the implants could carry sciatic nerve compression as a complication. For this reason, we prefer the intramuscular technique. Moreover, in their practice biconvex implants are chosen with possibility of flipping of the prostheses. In our practice, implants are round-shaped and in our opinion, this allows for a steadier result. Cárdenas-Camarena and Paillet were the first surgeons to combine gluteal implants positioning and liposuction [17]. They conducted a retrospective review on patients who underwent surgery for gluteal area improvement. The study included and analyzed all those who required gluteal implant placement and liposuction of adjacent areas to achieve integral enhancement of the area. The ideal patients for this combined technique were slim patients, with marked gluteal hypoplasia and little fat excess, principally in the supragluteal area. Liposuction accurately delimited the gluteal area and, to a certain degree, allowed a dissembling of the lack of projection, which was then completed with the placement of the gluteal implant. The authors considered this procedure safe and effective for the improvement of the gluteal profile, but it required keeping certain premises in mind. Adding liposuction produced a larger projection than gluteal implants placed without it. For this reason, some patients have felt that the implant was larger than expected, which had to be considered when deciding on size. Moreover, they tried to avoid possible predictable complications, implant displacement and appearance, seromas and hematomas formation, and to produce widely satisfactory results. A subsequent interesting article by Cárdenas-Camarena et al. [18] evaluated gluteal augmentation performed by combining buttock implant placement, frame liposuction, and lipoinjection in the lateral third of the buttock and on a plane superficial to the gluteal implant. They called this technique “tridimensional combined gluteoplasty.” They used anatomic-shaped cohesive silicone implants, with the most used size being 300 cc. The average volume of infiltrated fat in each hip and buttock was 243.1 cc and 141.6 cc respectively. As mentioned before we used round-shaped implants (sizes ranging from 225 to 440 cc), and harvest fat graft from the lumbar region to be injected in the area over the implants. This can help to create a more natural-looking and smooth appearance and can also help to improve the overall shape and contour of the buttocks. The fat is typically taken from an area with excess fat, such as the lumbar region, and is carefully processed before it is injected into the buttocks. The use of fat grafting in our experience can help to create a more aesthetically pleasing result and can also help to reduce the risk of complications. Godoy and Munhoz [19] believed that associating fat injection in the subcutaneous plane provided a more natural outcome. Their work described gluteal lipofilling safely performed over the implant, which is protected by the intramuscular plane, yielding firm and even projection, and the grafted fat disguises its superior portion. As regards lipoaspiration, they perform hips and flank area aspiration of fat, while we perform fat aspiration from the lumbar area only. This procedure guarantees, in our opinion, a better aesthetic result and shaping of body contour. In Godoy and Munhoz’s work, most of the patients were thin, with a body mass index below 20. Despite this fat volume limitation, adequate fat volumes could be found in the hip and flank areas. Ideal candidates are slim and have poor soft tissue coverage in the gluteal area. For example, they perform a pinch test, and when it results < 2 cm, they can successfully treat it with intramuscular implants associated with lipofilling. The authors have found that associated lipofilling has not been necessary for patients with a pinch test result greater than 2–3 cm; this amount of fat is considered a relative contraindication to their technique and an indication for conventional intramuscular gluteal augmentation. We do not usually follow this indication and do not routinely perform pinch tests to select patients undergoing lipoaspiration. Some potential advantages of performing gluteal augmentation under TLA include reducing respiratory depression and other drug-related complications. TLA typically uses a combination of local anesthetics and sedatives, which can help reduce the risk of complications associated with using more potent anesthetic drugs such as Propofol, Ketamine, or Fentanest. Moreover, performing gluteal augmentation under TLA has important advantages, such as shorter recovery time. This can allow a quicker return to daily activities for our patients. This technique also reduces the risk of nausea and vomiting. Some patients experience nausea and vomiting after surgery, especially after general anesthesia. TLA can help to reduce this risk, as the medications used during the procedure are less likely to cause these side effects. Overall, patient satisfaction is enhanced: the patient remains awake and responsive during surgery, which makes them feel more in control and less anxious. We have been able to use TLA on myasthenic patients, for whom general anesthesia usually carries higher complication rates. It is important to note that we strongly advise the presence of an anesthesiologist during the operation and patients should discuss the potential risks and benefits of TLA with the surgeon before undergoing gluteal augmentation. While it is true that epinephrine can cause vasoconstriction, leading to reduced blood flow and potentially less bleeding during surgery [20], it is essential to perform accurate hemostasis to avoid postoperative bleeding after the epinephrine effect ends. This can help to reduce the risk of complications such as excessive blood loss and the formation of hematomas. We recorded hematoma occurrence only in 2 patients (10%) and seroma formation in 1 patient (5%). This complication rate is comparable with the literature on intramuscular gluteal augmentation [14, 15], regardless of the type of anesthesia technique used. In addition, ensuring accurate hemostasis can help to minimize the risk of implant infection and the need to position aspiration drains. Avoiding drains can help to reduce the patient's discomfort and improve their overall surgical experience. In general, surgeons who have previously performed gluteal augmentation under general anesthesia will have a relatively easy time adjusting to the use of TLA for this procedure. This is because the surgical techniques involved in gluteal augmentation are generally similar regardless of the type of anesthesia used. One patient experienced muscular spasms after surgery and was promptly treated with a botulin toxin (BTX-A) injection [21]. Minor complications that we recorded were dystrophic scarring (1 case) and delay in wound closure (4 cases). We applied a polyurethane dressing in these cases to facilitate wound closure [22]. TLA allows early patient discharge and deambulation after a few hours, thus reducing the risk for deep venous thrombosis and with higher satisfaction and better comfort for patients that can rest and recover at home.

## Conclusion

TLA represents a safe and efficacious technique for performing gluteal augmentation surgery with implant positioning in an intramuscular pocket. This technique has proven to have a low incidence of postoperative side effects, with reasonable pain control throughout surgery and the immediate postoperative period. For these reasons, it is suitable for myasthenic patients and those people for whom general anesthesia is contraindicated. Patients were satisfied with the esthetic outcome and the pain management, and surgeons who have previously performed gluteal augmentation under general anesthesia will have a relatively easy time adjusting to the use of TLA for this procedure. A board-certified anesthesiologist is recommended for the correct selection of patients and in case of major anesthesia-related complications.
